# The Role of Quantitative EEG in the Diagnosis of Alzheimer’s Disease

**DOI:** 10.3390/diagnostics15151965

**Published:** 2025-08-05

**Authors:** Vasileios Papaliagkas

**Affiliations:** Department of Biomedical Sciences, International Hellenic University, 57400 Thessaloniki, Greece; vpapal@auth.gr

**Keywords:** qEEG, spectral analysis, Alzheimer’s disease

## Abstract

Alzheimer’s disease is the most prevalent neurodegenerative disorder leading to progressive cognitive decline and functional impairment. Although advanced neuroimaging and cerebrospinal fluid biomarkers have improved early detection, their high costs, invasiveness, and limited accessibility restrict universal screening. Quantitative electroencephalography (qEEG) offers a non-invasive and cost-effective alternative for assessing neurophysiological changes associated with AD. This review critically evaluates current evidence on EEG biomarkers, including spectral, connectivity, and complexity measures, discussing their pathophysiological basis, diagnostic accuracy, and clinical utility in AD. Limitations and future perspectives, especially in developing standardized protocols and integrating machine learning techniques, are also addressed.

## 1. Introduction

Alzheimer’s disease (AD) is the most prevalent neurodegenerative disorder that is responsible for sixty to seventy percent of all dementia cases [1]. AD creates a substantial personal and economic impact on society because the disease affects the aging population, causing its prevalence to rise dramatically in upcoming years [2]. The condition starts with subtle signs which develop into steady deterioration of mental functions primarily affecting memory, together with executive abilities, language skills, and spatial orientation. The disease progression leads people to lose their independence while developing behavioral problems which eventually result in total dependence.

### 1.1. Pathology and Progression of Alzheimer’s Disease

The major neuropathological hallmarks of AD are amyloid plaques that consist of b-amyloid as well as neurofibrillary tangles that consist of the hyperphosphorylated form of tau-protein [3]. The disease development creates synaptic impairment, neuronal damage, and network disruptions primarily in the hippocampus and temporal, parietal, and frontal cortices [4]. The disease process has a long preclinical phase which extends from several years to multiple decades until clinical symptoms become visible while neurodegeneration occurs without symptoms. The extensive preclinical period emphasizes the immediate requirement for trustworthy biomarkers which can detect initial pathological changes before dementia symptoms develop. Early diagnosis enables medical professionals to start disease-modifying treatments, and it helps plan care resources while providing patients and their families with better decision-making abilities.

### 1.2. Current Diagnostic Criteria and Biomarkers

Medical professionals have traditionally diagnosed Alzheimer’s disease through neuropsychological evaluations together with observable cognitive symptoms [5]. These clinical tests demonstrate poor performance during the initial stages of the disease. The combination of MRI and PET imaging with amyloid and tau pathology tracers through neuroimaging techniques has improved diagnosis significantly [6]. The biomarker panel which includes Aβ42, total tau, and phosphorylated tau measurement in cerebrospinal fluid (CSF) has established itself as a valuable diagnostic tool that represents fundamental pathological mechanisms [7]. These diagnostic tools face multiple challenges, because they require expensive procedures that are both invasive and limited in their availability which prevents their use in routine screening and population-wide screening.

In recent years, research has focused on the development of blood-based biomarkers for AD, offering a less invasive and more accessible alternative. Several promising biomarkers have emerged, including the plasma Aβ42/Aβ40 ratio, phosphorylated tau (p-tau) isoforms (e.g., p-tau181, p-tau217, and p-tau231), and glial fibrillary acidic protein (GFAP) [8]. Studies have shown that these blood-based biomarkers can differentiate between AD and healthy controls, predict the progression from mild cognitive impairment (MCI) to AD and correlate with amyloid and tau pathology in the brain [9,10]. While blood-based biomarkers hold great promise for early detection and diagnosis of AD, further research is needed to validate their accuracy, standardize assays and determine their optimal use in clinical practice.

### 1.3. Electroencephalography (EEG): A Non-Invasive Window into Brain Function

EEG functions as a neurophysiological assessment which detects brain electrical signals through scalp electrodes. EEG emerged during the early 20th century and medical professionals have utilized it extensively to analyze brain functions in healthy subjects as well as diseased patients [11]. The main approach to EEG analysis involves the study of certain wave patterns along with the identification of epileptic or encephalopathic pattern. The development of computational methods enables qEEG analysis through spectral and functional connectivity and nonlinear feature extraction from EEG signals. These quantifiable biomarkers enable medical professionals to measure brain activity while assessing network integrity. Compared to traditional EEG that relies mainly on visual interpretation, qEEG provides objective indicators through linear and nonlinear analysis and can detect changes in neural synchronization, disconnection, and abnormal oscillation.

However, qEEG interpretation is not without its challenges. Factors such as patient age, alertness, medication use, and underlying medical conditions can influence EEG activity and complicate the interpretation process. Artifacts from muscle movement, eye blinks, and external electrical interference can also obscure the underlying brain signals [12].

To aid in EEG interpretation, computer-assisted review tools, such as Persyst, have been developed [13]. These systems provide automated analysis of EEG data, including artifact detection and removal, spectral analysis, and pattern recognition. While not AI or machine learning models, these tools can assist clinicians in identifying and quantifying EEG abnormalities, potentially improving the efficiency and reliability of EEG interpretation. Objective scoring sheets and standardized reporting formats are also being developed to promote consistency and reduce inter-rater variability in EEG interpretation [14].

### 1.4. Rationale for EEG in Alzheimer’s Disease

Research conducted during the previous twenty years has demonstrated that AD produces specific EEG patterns which include spectral slowing, reduced alpha and beta power, elevated delta and theta activity, and disrupted network connectivity [15,16]. Research has established that these brain changes show direct relationships to both cognitive deterioration and the progression of disease severity [17]. The appeal of EEG as a biomarker lies in its non-invasiveness, cost-effectiveness, portability, and high temporal resolution. EEG provides an ideal solution for early screening applications, longitudinal assessments, and therapeutic monitoring because of its portable nature, cost-effectiveness and high temporal resolution. Early diagnosis becomes possible because EEG detects functional brain alterations while structural damage remains invisible.

This review aims to summarize existing knowledge about EEG patterns in AD, present the most promising qEEG biomarkers which include spectral, connectivity, and complexity patterns, as well as highlighting future research directions which include machine learning integration, portable devices, and multimodal biomarker strategies.

## 2. Pathophysiological Basis of EEG Alterations in AD

It is necessary to understand the neurobiological processes that lead to EEG abnormalities in Alzheimer’s disease (AD) in order to correctly understand EEG results in clinical practice or research. The typical EEG findings of spectral slowing, together with network disruptions, reflect exactly the neuropathological and neurochemical alterations that occur during AD progression.

### 2.1. Neurodegeneration and Synaptic Dysfunction

The main feature of AD pathology is neurodegeneration which mainly affects the hippocampus, entorhinal cortex, and association cortices in later stages [18]. The first phase of Aβ plaques and tau neurofibrillary tangles development causes continuous synaptic loss and neuronal death [19]. The rate of cognitive decline in patients with AD is directly related to the extent of synaptic degeneration rather than the extent of neuronal loss, thus affecting the oscillatory activity and neural circuit integrity. The synaptic degeneration results in reduced neuronal synchronization mainly in the circuits that produce alpha and beta rhythms. The decrease in synaptic density leads to reduced efficiency of neuronal firing and communication, which causes spectral changes toward theta and delta bands. This condition is known as neural disconnection which indicates disrupted activity between brain networks.

### 2.2. Disruption of Large-Scale Neural Networks

The brain functions as an organized system that depends on three main network groups: the Default Mode Network (DMN), fronto-parietal control network, and limbic circuits. These networks support cognitive processes including memory, attention, and executive functions [20]. AD pathology mainly affects the nodes in these networks by damaging the posterior cingulate cortex, the precuneus, and medial temporal structures, thus leading to their functional disconnection. The findings from resting-state functional MRI (fMRI) studies show significant network coherence alterations which match with EEG measures of spectral slowing and reduced connectivity [21]. The degradation of these networks results in reduced region-to-region synchronization which is manifested as lower EEG coherence and phase-locking values. The brain loses its ability to coordinate and integrate information as a result of these changes, which leads to the AD typical cognitive deficits.

### 2.3. Neurochemical Disruptions and Their Impact on Oscillatory Activity

EEG rhythms are shaped by the neurochemical alterations that include the deficiencies in cholinergic, glutamatergic, and GABAergic systems [22].

- Cholinergic Deficits: The cholinergic system of the basal forebrain degenerates during the initial stages of AD, which leads to reduced cortical activation and reduced alpha rhythm activity, because alpha rhythms are generated by cholinergic projections through thalamo-cortical circuits [23]. Alpha power decreases due to reduced cholinergic tone, which leads to impaired attention and memory function.
- GABAergic Dysfunction: The changes in GABAergic interneurons result in impaired inhibitory−excitatory balance, which disrupts the generation of gamma oscillations that are necessary for higher cognitive functions [24].
- Glutamatergic Dysregulation: The excitotoxicity effects of glutamate on neurons and networks result in unstable network dynamics, which leads to both spectral slowing and abnormal synchrony patterns.

### 2.4. Neuroinflammation and Gliosis

The recent scientific findings show that neuroinflammation plays an essential role in the progression of AD. Microglia and astrocyte activation results in cytokine secretion and oxidative stress which further damages neurons [25]. This inflammatory environment changes how neurons work and how synapses function, which makes the spectral slow down and network disintegration worse.

### 2.5. Linking Pathology to EEG Manifestations

The combination of these pathophysiological processes creates a direct connection between molecular and histopathological changes and the observed EEG changes, such as the following:

A. Spectral Slowing: The combination of synaptic and neuronal loss, disrupted reciprocating networks, and neurochemical deficits leads to delta and theta power increase and alpha and beta power decrease.
B. Connectivity Disruption: The extensive breakdown of brain networks results in reduced phase synchronization in the alpha frequency band which impairs cognitive processes.
C. Reduced Complexity: The loss of neurons and synaptic connections decreases the dynamical complexity of EEG signals which can be measured by entropy and fractal dimension.

EEG changes in AD represent core pathological functions that create functional signatures, which reveal the progressive deterioration of essential cognitive neural networks.

## 3. EEG Biomarkers in AD: Spectral, Connectivity, and Complexity Measures

EEG studies in AD have moved nowadays from the basic visual inspection of waveforms to the use of advanced quantitative methods which analyze spectral patterns, functional dynamics, and nonlinear behavioral aspects. These biomarkers provide unique windows into the disease’s neurobiological impact on brain function.

### 3.1. Spectral Power Measures

The EEG signal decomposition process through spectral analysis reveals neural oscillation patterns which correspond to cortical activity states. Studies show consistent spectral slowing patterns in AD patients, which demonstrates both neurodegenerative processes and disintegrating network structures.

#### 3.1.1. Alpha Band (8–13 Hz) Reduction

Thalamo-cortical circuits generate alpha oscillations during restful periods with eyes closed [26]. Alpha waves are essential for processes that include attention and sensory processing along with cortical resting state activity. Several research studies have documented significant alpha power reductions primarily affecting occipital, parietal, and posterior temporal brain areas in AD patients [27]. The reduction in alpha power in patients directly corresponds to the severity level of cognitive deterioration and hippocampal damage. The cause of the alpha rhythm reduction in AD patients is believed to be the deterioration of the cholinergic pathways while thalamic relay neurons malfunction and synaptic connections disappear.

#### 3.1.2. Theta Band (4–8 Hz) Elevation

The brain shows strong theta activity throughout the medial temporal and frontal areas of AD patients. Memory deficits, together with reduced cognitive performance, show strong correlation with elevated theta activity [28]. The activity of the hippocampus generates theta oscillations that enable memory encoding and other cognitive processes. The hippocampal damage in AD causes an increase in theta activity because cortical circuits experience disrupted inhibition and disinhibition processes [29].

#### 3.1.3. Delta Band (0.5–4 Hz) Enhancement

AD patients demonstrate higher relative power spectrum density in the delta frequency band [30]. It is believed that enhanced delta activity reflects cortical deafferentation and global neuronal network disintegration.

#### 3.1.4. Beta (>13 Hz) and Gamma (>30 Hz) Bands Decreases

The brain uses high-frequency oscillations as an indicator for active cognitive processing. The power of beta and gamma frequencies shows significant reduction in AD patients throughout prefrontal and temporal brain regions. Research indicates that these power reductions contribute to the deterioration of attention capabilities and executive functions along with working memory abilities [31]. The impairment of interneuron networks, together with neurochemical deficits, results in reduced gamma oscillations which are fundamental to cognitive processes.

#### 3.1.5. Spectral Ratios and Composite Indices

**Figure 1:**
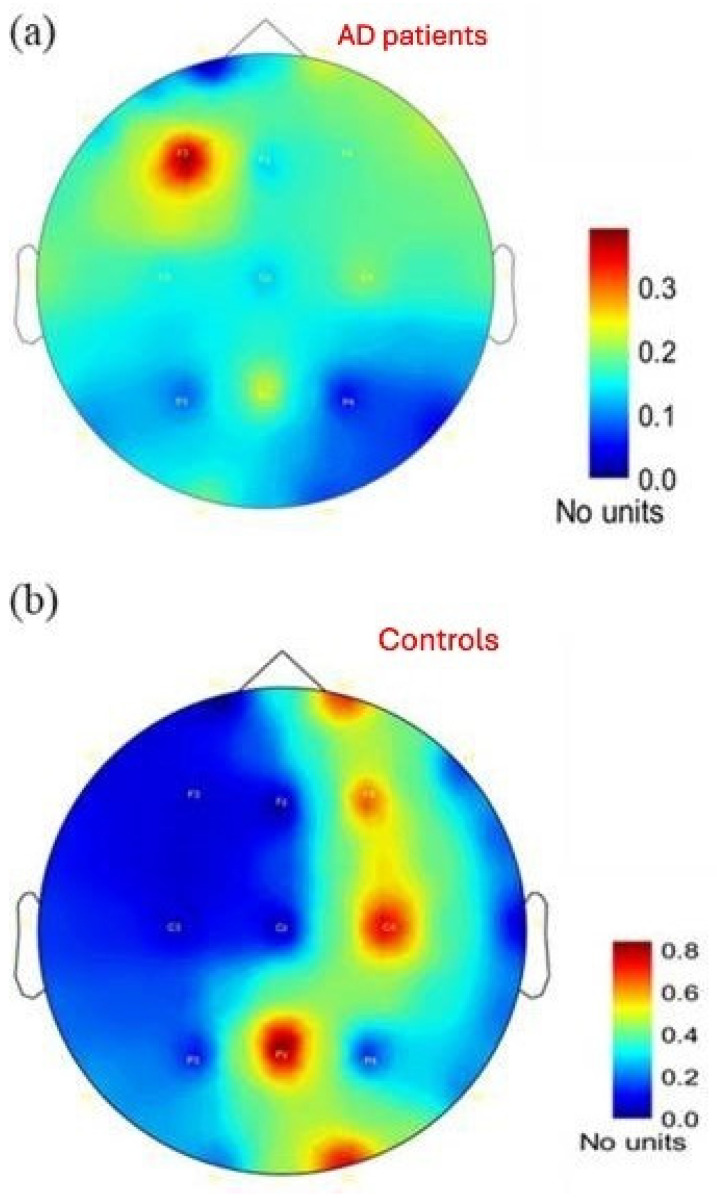
Power Spectral Density Map for (**a**) AD patients and (**b**) Controls. Figure adapted by Simfukwe C et al. [32], CC License 3.0.

The computation of theta/alpha and theta/(alpha + beta) ratios enhances the pattern of slowing in brain activity. Early diagnostic accuracy exceeds absolute power measures when using these specific ratios [32]. The theta/alpha ratio elevation demonstrated potential to forecast the transition of MCI patients to AD [33]. Figure 1 depicts power spectral density topographic maps in AD patients and healthy controls.

### 3.2. Connectivity Measures

Connectivity metrics help researchers observe how different brain regions work together to coordinate their activities, which reveals information about network integrity.

#### 3.2.1. Functional Connectivity: Coherence and Phase-Based Metrics

The analysis of coherence evaluates the linear relationship between signals at their particular frequencies. In AD, it was observed that alpha and beta coherence decreases significantly between parietal, temporal, and frontal regions, which demonstrates impaired communication pathways [34]. This network connectivity failure directly impacts cognitive performance by affecting memory processing and executive function abilities.

#### 3.2.2. Network Topology via Graph Theory

Graph theoretical measures quantify the architecture of brain networks. In particular, the clustering coefficient decreases in AD, indicating less local clustering, while the characteristic path length increases, reflecting longer or impaired information transfer paths. The network measures indicate reduced global and local efficiency, which demonstrates inferior network integration [30]. Moreover, network structures change toward less efficient small-world patterns and more random configurations that match neurodegenerative disease patterns.

#### 3.2.3. Directed and Causal Connectivity

AD leads to interrupted neural pathways that mainly affect information transmission between hippocampal and posterior association cortices and prefrontal areas. The research backs up the theory, which shows Alzheimer’s disease creates impairment of brain hierarchical organization and information integration needed for memory function and executive tasks [35]. Altered brain connectivity patterns serve as specific markers which help identify disease stages and monitor disease progression. The combination of spectral features with these markers allows for better differentiation between various neurodegenerative diseases for purposes of diagnosis.

### 3.3. Nonlinear and Complexity Measures

The brain demonstrates complex nonlinear dynamical processes which surpass basic linear spectral and connectivity measurements. The recurrent neural activity gives brains adaptability, robustness, and information processing abilities which both decline during AD development. Research indicates Alzheimer’s disease patients have lower sample entropy measurements, which reveals reduced neural complexity together with decreased adaptive functions [36]. On the other hand, studies have shown AD patients present with reduced approximate entropy scores that link to worsening cognitive performance [37].

#### 3.3.1. Fractal and Multiscale Analysis

- Hurst exponent and fractal dimension: EEG signals receive evaluation through these measures for their temporal correlations and self-similar patterns. AD patients exhibit decreased fractal dimension values that point to more organized, less complex brain activity patterns [38].
- Multiscale entropy (MSE): The method shows complexity patterns across different time frames that show AD patients have lower values than healthy controls [39].

#### 3.3.2. Chaos and Lyapunov Exponents

The measurement of Lyapunov exponents reveals brain system sensitivity to initial conditions, which indicates chaotic processes in the brain that are directly linked to neural networks and neurological diseases. These processes are observed in Alzheimer’s disease and other dementias, and due to their complex and seemingly random behavior, they may be analyzed by EEG signals. In particular, EEG signals from AD patients show lower Lyapunov exponents, which indicates a decrease in system flexibility along with diminished dynamical variability [40]. The diminished markers of entropy and fractal dimension and chaos indicators match essential disease characteristics which include neuronal loss, synaptic disconnection, and network disintegration. Nonlinear biomarkers show significant correlations with cognitive scores that makes them useful tools to monitor disease progression and evaluate treatment effects.

### 3.4. Machine Learning Models

The use of machine learning (ML) in dementia research has grown quickly by incorporating various data types, including MRI positron emission tomography (PET), qEEG, genetic biomarkers, speech patterns, and electronic health records (EHRs). A broad spectrum of ML algorithms, including support vector machines, random forests, deep learning architectures, and ensemble methods, have been developed and evaluated for their diagnostic performance and combined with qEEG. The robustness of support vector machines (SVMs) in handling high-dimensional spaces makes them suitable for neuroimaging and qEEG-based dementia classification. Linear SVMs work well for basic linearly separable problems, but kernel-based SVMs are needed to model the nonlinear relationships that are typical in complex clinical data. They work well with small to moderate-sized datasets but may not perform well with large-scale, multimodal inputs unless dimensionality reduction is applied first. In particular, Bairagi [41] used spectral features and wavelet features together with SVM for AD diagnosis to diagnose AD. That was achieved with an accuracy of 94%, which is significantly better than the results of using spectral features (90%) or wavelet features (88%) alone. ML models have shown promise in improving the accuracy of MCI and AD diagnosis by identifying complex patterns in qEEG data that may not be readily apparent through traditional visual inspection or conventional statistical analysis. For instance, ML algorithms can be trained to classify individuals as having MCI, AD, or normal cognition based on spectral power, connectivity, and complexity measures extracted from qEEG recordings [12].

Moreover, ML models can also be used to predict the likelihood of MCI patients converting to AD. By incorporating longitudinal qEEG data, along with other clinical and demographic variables, these models can identify individuals at high risk of developing AD, allowing for earlier intervention and enrollment in clinical trials [13]

ML models could also be implemented to predict individual responses to AD treatments, such as cholinesterase inhibitors or emerging disease-modifying therapies. By analyzing pre-treatment qEEG data, ML algorithms can identify patterns that are associated with a greater likelihood of response to a particular treatment [14] and can help predict the rate of cognitive decline and disease progression, providing accurate prognoses, facilitating patient care planning, and resource allocation.

Moreover, deep learning techniques which utilize Convolutional Neural Networks (CNNs) for imaging data and Recurrent Neural Networks (RNNs) or Long Short-Term Memory (LSTM) networks for time-series or longitudinal data. These demonstrate success in tracking cognitive decline progression through sequential clinical or qEEG data, as well as by integrating longitudinal qEEG data with other clinical and biomarker information [42].

## 4. Clinical Utility and Diagnostic Value

Multiple research studies conducted throughout the past two decades demonstrate that quantitative EEG (qEEG) biomarkers show strong potential to improve clinical care for AD The non-invasive EEG measures provide both cost-effective and accessible observations of neural dysfunction, which support diagnosis in early stages as well as tracking disease progression and treatment response. The following discussion examines essential uses of EEG-based measurements along with their supporting research and both their benefits and restrictions.

### 4.1. Early Detection and Prodromal Diagnosis of MCI and AD

EEG biomarkers enable the identification of functional changes before noticeable clinical signs become apparent. Studies have shown that individuals with MCI often exhibit distinct EEG characteristics compared to healthy controls, including altered spectral power (e.g., increased theta power and decreased alpha power) and changes in brain network connectivity [43,44,45]. These EEG changes may reflect early neuronal dysfunction and can potentially aid in the identification of individuals at risk for cognitive decline. Research conducted over time demonstrates that spectral slowing with increased theta power and higher theta/alpha ratios appears in patients with MCI who develop AD [46,47]. EEG biomarkers like decreased alpha power together with increased delta/theta activity demonstrate high predictive accuracy for MCI patients who will develop AD with success rates above 80% in two separate studies [48,49]. The results demonstrate consistent results in multiple research studies, which strengthens their potential as initial diagnostic markers. A summary of qEEG studies for MCI and AD diagnosis that include the qEEG measures examined and their diagnostic accuracy, sensitivity, and specificity is presented in Table 1.

qEEG may serve as a scalable screening instrument for vulnerable groups in primary care facilities and community centers, particularly when advanced neuroimaging and CSF analysis are challenging to access in limited resource settings.

### 4.2. Differential Diagnosis of Dementias

The clinical features of AD overlap with vascular dementia as well as Lewy body dementia and frontotemporal dementia (FTD). Accurate differentiation is critical for prognosis, management, and inclusion in clinical trials. EEG patterns in AD patients show posterior spectral slowing, together with decreased alpha activity and disrupted synchronization. The EEG patterns of vascular dementia show focal and asymmetric changes, which differ from the typical posterior slowing of FTD and the occipital alpha patterns found in Lewy body dementia patients with visual hallucinations [59].

### 4.3. Disease Staging and Progress Monitoring

EEG biomarkers enable medical professionals to monitor disease progression because spectral slowing and network disintegration show direct correlations with worsening cognitive performance and reduced functional autonomy. Research indicates that increases in theta/alpha ratios and global efficiency reduction serve as predictors for mild cognitive impairment patients to develop overt Alzheimer’s disease [60]. The progression of EEG slowing patterns directly corresponds with worsening MMSE and ADAS-Cog scores as well as other neuropsychological assessment results [61]. These assessments help determine disease stages for better selection of treatment timing and individual patient care strategies.

### 4.4. Evaluating Therapeutic Efficacy and Response

EEG biomarkers show high sensitivity to therapeutic effects from pharmacological treatments and non-pharmacological interventions, thus making them useful treatment response indicators. The use of cholinesterase inhibitors such as donepezil and rivastigmine leads to small increases in alpha power and connectivity improvements that correlate with stable cognitive function or slight cognitive enhancement [62,63]. EEG changes allow objective measurement of neuronal network modulation despite the presence of clinical variations among patients. Moreover, research indicates that transcranial magnetic stimulation (TMS) and transcranial direct current stimulation (tDCS) produce short-term benefits by normalizing spectral markers and improving connectivity, which leads to cognitive improvements [64].

### 4.5. Addressing Challenges and Moving Forward

The successful deployment of EEG biomarkers for clinical use requires multiple stakeholders to work together. While qEEG shows promise as a potential tool for AD management, it is crucial to acknowledge that EEG biomarkers are not yet fully validated for widespread clinical use. Achieving this validation requires addressing key challenges that will be mentioned in this section. Firstly, the development of standardized EEG data acquisition, preprocessing, and feature extraction methods needs international consensus for both clinical applications and AD research settings [65]. The creation of detailed normative databases which include age and sex factors and cognitive status variables will help standardize interpretation procedures [66]. The platform should support data exchange between different research centers to validate algorithms while establishing common performance standards. In particular, EEG data from well-characterized AD and control cohorts against these normative values to determine the sensitivity and specificity of EEG biomarkers for different stages of the disease should be collected. Explainable AI techniques need investment for understanding the neural features that neural networks use to make classifications, which will build clinical trust; thus, the validation of these methods must include comparisons from expert electroencephalographers in the field in order to ensure that their reports are comparable.

The integration of EEG biomarkers should occur within multimodal diagnostic systems which combine neuroimaging data with fluid biomarkers to produce a complete diagnostic method. The hardware requires investment to become portable while becoming more affordable, thus allowing widespread usage particularly in primary care settings and community screening programs.

## 5. Discussion

Multiple research findings demonstrate that quantitative EEG (qEEG) biomarkers show great promise as non-invasive cost-effective diagnostic tools which help identify Alzheimer’s disease stages and manage its treatment. The neurophysiological changes detected through EEG match the disease progression and functional impairment of AD because they include spectral slowing, connectivity disturbances, and neural complexity reduction.

### 5.1. Neurobiological Significance of EEG Alterations

The neurobiological processes that lead to synaptic degeneration, neuronal loss, and network disconnection result in the spectral slowing phenomenon. The slowing pattern stems from damaged thalamo-cortical circuits, together with broken hippocampal function and disintegration of brain networks. Alpha oscillation decreases that match cortical idling and attentional processes reflect cholinergic deficits and thalamic dysfunction which serve as central mechanisms in AD pathophysiology [67]. Delta and theta activity increases serve as actual indicators of deafferentation while showing signs of cortical disconnection and neurodegenerative processes. The electrophysiological changes match the MRI findings of structural atrophy and post-mortem pathological burden, thus establishing a connection between functional changes and molecular mechanisms.

### 5.2. Diagnostic and Prognostic Utility

AD detection in its preclinical stages represents the essential objective of biomarker investigation. Research indicates that elevated theta/alpha ratios in spectral markers show high effectiveness in identifying AD converters from MCI before noticeable cognitive deterioration sets in [68]. Moreover, as mentioned in Table 1, other power spectral density and functional connectivity measurements can diagnose AD and MCI compared to normal controls with sensitivity and specificity that may even reach over 90% [41,51]. These findings suggest that EEG can capture subtle functional changes occurring before significant cognitive decline becomes apparent, offering a potential window for early intervention. While spectral slowing is a common feature, the specific patterns of slowing and connectivity disturbances appear to be distinct across different dementia subtypes [58]. It seems that focal slowing may indicate vascular contributions, whereas more widespread slowing may indicate neurodegenerative processes. These findings underscore the importance of a comprehensive EEG analysis and cannot be derived by a simple visual inspection. As described in Section 4, the diagnostic specificity of EEG-based biomarkers increases when combined with neuropsychological assessments. The author believes that integrating EEG data with other biomarkers such as blood-based biomarkers, neuroimaging, and neuropsychological assessment can provide a more comprehensive picture of disease progression and treatment efficacy.

### 5.3. Challenges and Limitations

Several obstacles prevent EEG biomarkers from being translated directly into clinical practice at present:

(a) The absence of standardized methods for acquiring data and processing EEG signals through different protocols, electrode placements, and feature extraction methods creates challenges for achieving universal diagnostic criteria. The interpretation of clinical cases becomes more difficult because of inconsistent data and insufficient normative database availability.
(b) EEG signals remain susceptible to artifacts from physiological and environmental sources which require advanced processing methods and skilled expert evaluation that most clinical settings cannot provide.
(c) Patient populations exhibit diverse characteristics because vascular diseases, depressive disorders, and medication side effects make it difficult to determine if EEG results stem from Alzheimer’s disease.
(d) The majority of research uses small, cross-sectional study populations; extensive multicenter longitudinal validation studies must be conducted to establish clinical thresholds and generalizability. This limitation was mentioned in many qEEG studies [53,57,58].

### 5.4. Opportunities for Future Research and Clinical Integration

The complete diagnostic utility of EEG requires additional advancements. The adoption of EEG for diagnosis requires standardization of protocols for EEG recording, preprocessing, feature extraction, and reporting according to international consensus guidelines. The databases should establish age-based and sex-based parameters along with health conditions for normative data. The creation of an enhanced diagnostic system demands the combination of EEG data with structural MRI and PET imaging, CSF biomarkers, and genetic profiling. Explainable AI development should continue to enhance both physician trust and regulatory acceptance of AI-based systems. User-friendly portable devices are needed to support EEG testing in multiple locations including healthcare facilities and personal residences. The combination of repeated neurophysiological assessments of individual patients through machine learning algorithms enhances disease detection capabilities while improving prediction results over time.

## 6. Conclusions

The use of quantitative EEG (qEEG) shows promise for developing early non-invasive biomarkers which diagnose Alzheimer’s disease (AD) effectively, although they are not included in the diagnostic criteria. The neurodegenerative process in Alzheimer’s disease causes functional brain changes, such as reduced neural complexity, spectral slowing, and connectivity disruption, which EEG methods detect effectively. The high temporal resolution, portability, and relatively low cost of EEG make it an attractive tool for widespread screening, disease staging, and longitudinal monitoring. The medical field has used EEG biomarkers to diagnose and predict disease progression in people with mild cognitive impairment (MCI) and prodromal stages during the last several decades. The combination of spectral and functional connectivity, along with nonlinear dynamic measures, provides multiple dimensions to study the complex neurophysiological changes in AD.

EEG biomarkers face major obstacles to achieve routine implementation in medical practice. Standardization efforts for acquisition and analysis protocols need improvement, and large diverse cohort validation is necessary as well as the development of normative databases and user-friendly analytical tools with explainable features. The resolution of these obstacles needs researchers from neuroscientific fields to work together with clinical specialists, engineering experts, and policy-making professionals. The future will bring rapid clinical adoption because of advancements in machine learning algorithms together with portable EEG hardware and multimodal biomarker integration. The reliability and robustness of EEG-based diagnostics will become established through international standards, large-scale multicenter studies, and continuous validation. EEG-based biomarkers provide an efficient method to detect Alzheimer’s disease at an early stage while tracking disease progression along with therapeutic outcome assessments. Their complete utilization depends on proper validation alongside standardization initiatives and technological breakthroughs.

Therefore, future research should focus on the following:

1. Systematic Review and Meta-Analysis of EEG Spectral Power for MCI Subtype Differentiation and AD Prediction: A rigorous systematic review and meta-analysis is needed to quantify the diagnostic accuracy of specific EEG spectral power changes (e.g., increased theta and decreased alpha) in differentiating amnestic MCI (aMCI) from non-amnestic MCI (naMCI). This review should analyze inter-study heterogeneity (e.g., variations in EEG acquisition protocols and subject populations) and assess the predictive power of these spectral features for conversion to AD, considering factors like APOE ε4 status. This review should explicitly address the limitations of existing studies, such as small sample sizes and lack of longitudinal data.
2. Well-designed, double-blind RCT should evaluate the efficacy of personalized EEG-neurofeedback therapy for individuals with early-stage AD (or prodromal AD). The intervention protocol should be tailored based on individual EEG profiles (e.g., targeting specific spectral imbalances). The trial should include a clearly defined control group (e.g., sham neurofeedback) and objective cognitive outcome measures (e.g., ADAS-Cog) and assess changes in functional connectivity using EEG. Power analysis should be performed to determine the appropriate sample size.
3. Multicenter Longitudinal Study of EEG and Blood-Based Biomarkers for AD Risk Stratification: A multicenter, longitudinal study should investigate the combined utility of EEG and emerging blood-based biomarkers (e.g., plasma p-tau isoforms and GFAP) for risk stratification in individuals with subjective cognitive decline (SCD). This study should employ standardized EEG acquisition and analysis protocols across all sites and collect longitudinal data on cognitive function, biomarker levels, and clinical outcomes. The study should also address potential confounding factors, such as vascular risk factors and medication use.
4. Development and Validation of Explainable AI Algorithms for EEG-Based AD Diagnosis: Research should focus on developing and validating explainable AI (XAI) algorithms for EEG-based AD diagnosis. These algorithms should not only provide accurate classifications but also offer transparent explanations of the EEG features driving their predictions, enhancing clinician trust and facilitating clinical integration.

The successful implementation of EEG biomarkers in clinical practice would produce significant improvements for patients through early diagnosis and individualized care, which ultimately decreases the impact of Alzheimer’s disease on society.

## Figures and Tables

**Table 1:** qEEG studies for the diagnosis of mild cognitive impairment and Alzheimer’s disease.

Study Sample (Patients)	qEEG Parameters Studied	Results	References
AD 50, NC 57	Alpha/theta ratio	Discrimination between AD and normal controls (NCs): sensitivity = 73%; Specificity = 82%	Schmidt et al., 2013 [50]
AD 50, NC 50	Spectral and wavelet	Discrimination between AD and NC: sensitivity = 92–96%; specificity = 84–92%	Bairagi, 2018 [41]
11 MCI, 17 NC	Wavelet transformation−ELM-based model	Discrimination between MCI and NC:	Siuly et al. [51]
		sensitivity = 98.32% and specificity = 99.66%	
ADMCI 30, DLBMCI 23, NC 30	eLORETA	ADMCI vs. NC: sensitivity = 90.0%; specificity = 73.3%	Babiloni et al. [52]
AD 13, bvFTD 13NC 18	Functional connectivity analysis	AD vs. NC: AUC = 0.54; Acc = 44.9%	Dottori et al. [53]
AD 63, MCI 63, NC 63	PSD image and spectral features	AD vs. MCI vs. NC: accuracy = 83.33%	Ieracitano et al. [54]
AD 20, NC 20	Functional connectivity analysis	AD vs. NC: accuracy = 84.7%; sensitivity = 86.2%; specificity = 83.2%	Klepl et al. [55]
AD 18, MCI 18, NC 18	Frequency-domain features	AD vs. MCI: 88.9% sensitivity and 72.2% specificity; AD vs. NC: 89% sensitivity and 77.8% specificity	Gkenios et al. [56]
AD 20, NC 20	Functional connectivity analysis and power spectral density	AD vs. NC: accuracy = 89.1%	Klepl et al. [57]
AD 330, MCI 189, NC 246	Hjorth metrics and sample entropy	AD vs. MCI vs. NC: diagnostic accuracy = 70%	Jiao et al. [58]
